# The Relevance of Transcription Factors in Gastric and Colorectal Cancer Stem Cells Identification and Eradication

**DOI:** 10.3389/fcell.2020.00442

**Published:** 2020-06-18

**Authors:** Diana Pádua, Paula Figueira, Inês Ribeiro, Raquel Almeida, Patrícia Mesquita

**Affiliations:** ^1^i3S – Institute for Research and Innovation in Health, University of Porto, Porto, Portugal; ^2^Institute of Molecular Pathology and Immunology, University of Porto, Porto, Portugal; ^3^Faculty of Medicine, University of Porto, Porto, Portugal; ^4^Department of Biology, Faculty of Sciences, University of Porto, Porto, Portugal

**Keywords:** gastric cancer, colorectal cancer, transcription factors, cancer stem cells, signaling pathways, targeted therapy

## Abstract

Gastric and colorectal cancers have a high incidence and mortality worldwide. The presence of cancer stem cells (CSCs) within the tumor mass has been indicated as the main reason for tumor relapse, metastasis and therapy resistance, leading to poor overall survival. Thus, the elimination of CSCs became a crucial goal for cancer treatment. The identification of these cells has been performed by using cell-surface markers, a reliable approach, however it lacks specificity and usually differs among tumor type and in some cases even within the same type. In theory, the ideal CSC markers are those that are required to maintain their stemness features. The knowledge that CSCs exhibit characteristics comparable to normal stem cells that could be associated with the expression of similar transcription factors (TFs) including SOX2, OCT4, NANOG, KLF4 and c-Myc, and signaling pathways such as the Wnt/*β*-catenin, Hedgehog (Hh), Notch and PI3K/AKT/mTOR directed the attention to the use of these similarities to identify and target CSCs in different tumor types. Several studies have demonstrated that the abnormal expression of some TFs and the dysregulation of signaling pathways are associated with tumorigenesis and CSC phenotype. The disclosure of common and appropriate biomarkers for CSCs will provide an incredible tool for cancer prognosis and treatment. Therefore, this review aims to gather the new insights in gastric and colorectal CSC identification specially by using TFs as biomarkers and divulge promising drugs that have been found and tested for targeting these cells.

## Introduction

Gastrointestinal malignancies are listed among the major causes of cancer death worldwide, being associated with environmental and genetic risk factors such as older age, chronic inflammation, family history, smoking, dietary patterns, overweight and physical inactivity, as well as gut microbiota (Lochhead and El-Omar, 2008; Mattiuzzi et al., 2019). Gastric cancer (GC) and colorectal cancer (CRC) are among the top five most incident and deadly cancers worldwide (Bray et al., 2018). As surgical techniques improve, as well as radiotherapy, chemotherapy and neoadjuvant therapies, the 5-year survival rate can reach up to 95% for early GC and 90% for localized CRC (Song et al., 2017; Sonbol et al., 2019). However, most patients have advanced-stage disease at diagnosis and so the best surgical window is missed (Song et al., 2017; Bray et al., 2018). They develop recurrent loco-regional disease or distant metastases with consequent decrease in survival. The heterogeneity of cancer at molecular, histological and phenotypic levels plays an important role in therapy resistance and tumor recurrence, being cancer stem cells (CSCs) among the major causative factors of cancer heterogeneity and treatment failure (Iseghohi, 2016; Gullo et al., 2018). The CSC model of tumor progression hypothesizes that a small subpopulation of cancer cells that display stem-like properties sustains tumor growth, metastasis, relapse and resistance to chemotherapy (Iseghohi, 2016). CSCs can undergo symmetric and asymmetric divisions, having the ability to give rise to all the different types of cancer cells within the tumor (Marjanovic et al., 2013). The origin of CSCs is still unclear and controversial (Dalerba et al., 2007a; Brunner et al., 2012). Various hypotheses suggest that depending on the tumor type, CSCs might be derived from adult stem cells, adult progenitor cells that underwent mutations, or differentiated cells that gained stem-like properties through dedifferentiation (Basu et al., 2016; Phi et al., 2018). A large number of studies demonstrate that CSCs share biomarkers with normal stem cells, thus specific markers for their identification have been explored in recent years. It is known that some transcription factors (TFs) can be re-expressed or reactivated in CSCs, playing a crucial role in the reprogramming of these cells. This review aims to provide a better understanding on how TFs associated with gastric and colorectal CSCs phenotype can be used for CSCs identification, characterization and targeted therapy.

## The Challenges of Cancer Stem Cell Identification

The identification of normal stem cells is now an easy process due to their well-recognized set of biomarkers whereas the identification of CSCs is a challenging task resulting from their complex phenotype, that differs from one tumor to another (Pattabiraman and Weinberg, 2014). Additionally, CSCs represent a very small percentage of tumor cells within the total tumor mass making even harder their detection in heterogeneous tumors (Kim Y. et al., 2009). The use of different combinations of cell-surface markers has been the main strategy to identify CSCs in several tumor types (Magee et al., 2012). The cell-surface markers are chosen according to their expression and relevance in the tumor type allowing the separation of CSCs from non-CSCs (Pattabiraman and Weinberg, 2014). In GC and CRC, the list of cell-surface markers capable of identifying CSCs is growing (Table 1). This means that the interest in gastric and colorectal CSCs identification and targeting is rising but also means that the already used markers are not uniformly advantageous for CSCs detection. In particular, inconsistencies remain concerning which cell-surface marker may be the ideal marker to identify gastric and colorectal CSCs from cell lines and primary tumors (Takaishi et al., 2009; Zhang et al., 2011; Jiang Y. et al., 2012; Rocco et al., 2012; Wakamatsu et al., 2012; Brungs et al., 2016).

**TABLE 1:** Cancer stem cell (CSC) biomarkers for gastric and colorectal cancer.

	**Gastric Cancer**	**Colorectal Cancer**
**CSCs biomarkers**	**References**	**References**
CD44	Takaishi et al., 2009; Zhang X. et al., 2016	Du et al., 2008; Huang et al., 2015
CD44 combined with CD24	Zhang et al., 2011	—
CD44 combined with CD54	Chen et al., 2012	—
CD44 combined with EpCAM	Han et al., 2011	Dalerba et al., 2007b
CD44 combined with CD133	—	Huang et al., 2015
CD49f	Fukamachi et al., 2013	Haraguchi et al., 2013
CD71	Ohkuma et al., 2012	—
CD90	Jiang J. et al., 2012	—
CD133	Fukamachi et al., 2011; Zhang X. et al., 2016	O’Brien et al., 2007; Ricci-Vitiani et al., 2007; Huang et al., 2015
CD133 combined with CD166	—	Dalerba et al., 2007b
CD184 (CXCR4)	Fujita et al., 2015	—
CD200	—	Zhang S.S. et al., 2016
Lgr5	Gong et al., 2016	—
Lgr5 combined with EpCAM	—	Kemper et al., 2012
Lgr5 combined with EpCAM and CD44	—	Leng et al., 2018
ALDH	Zhi et al., 2011	Huang et al., 2009
SOX2	Pádua et al., 2020	Takeda et al., 2018

*The table contains some of the most reported CSCs markers used for both tumor types.*

### Gastric Cancer

Several cell-surface markers have emerged for gastric CSC identification. The transmembrane glycoprotein CD44 was the first described cell-surface marker used in gastric CSC identification. Takaishi et al. (2009) and his collaborators analyzed a panel of six GC cell lines and in three of them – NCI-N87, MKN-45 and MKN-74 – a small CD44^+^ cell subpopulation displayed CSC features such as self-renewal, asymmetric division, spheroid colony formation, and *in vivo* tumorigenic ability. They also observed that the CD44^+^ subpopulation had a higher resistance to anticancer drugs when compared to CD44^–^ cells (Takaishi et al., 2009). However, in the other three cell lines – AGS, Kato III and MKN28 – the CD44 cell-surface marker was not able to mark cells with stem cell properties (Takaishi et al., 2009). Clinically, CD44^+^ cancer cells at the invasive GC front are associated with poor patient survival (Nosrati et al., 2014; Kodama et al., 2017). Later, Zhang et al. (2011) combined CD44 with CD24, a signal transducer, and successfully detected a CD44^+^CD24^+^ cellular subpopulation with CSCs characteristics, such as the capability to self-renew and to originate differentiated progeny (Zhang et al., 2011). Additionally, they showed that CD44^+^CD24^+^ cells had higher ability to form tumors when injected into immunodeficient mice, compared to the CD44^–^CD24^–^ cells (Zhang et al., 2011). The CD54 cell-surface marker, also known as ICAM-1 (intercellular adhesion molecule 1), was combined with CD44 to isolate gastric CSCs from tumor tissues and peripheral blood of patients with GC (Chen et al., 2012). The CD44^+^CD54^+^ cells exhibited *in vitro* and *in vivo* self-renewal ability, formed gastric tumorspheres and originated tumors similar to the original human tumor when injected into immunodeficient mice (Chen et al., 2012). The epithelial cell adhesion molecule (EpCAM) has also been used in combination with CD44 to mark gastric CSCs. The small EpCAM^+^/CD44^+^ subpopulation isolated from primary human GC tissues was more resistant to anticancer drugs including 5-fluorouracil (5-FU), doxorubicin, vinblastine and paclitaxel, when compared with EpCAM^+^/CD44^–^, EpCAM^–^/CD44^+^ and EpCAM^–^/CD44^–^ cells (Brabletz et al., 2005; Han et al., 2011). It also showed capacity to form sphere-like structures in serum free conditions and greater ability to originate tumors in immunocompromised mice (Han et al., 2011). The tumors formed after inoculation of the EpCAM^+^/CD44^+^ cells recapitulated the heterogeneous morphology and phenotype present in the original gastric tumor (Han et al., 2011). Moreover, Fukamachi et al. (2013) identified another potential gastric CSC marker, the CD49f, an integrin α6 (ITGA6) that is a subunit of laminin receptors. Their work showed that CD49f^+^ cells from GC originated tumors when subcutaneously injected into immunodeficient mice, while CD49f^–^ cells did not (Fukamachi et al., 2013). They also demonstrated that some of the CD49f^+^ sphere-forming cells were more resistant to doxorubicin, 5-FU and doxifluridine than the other GC cells studied (Fukamachi et al., 2013). Another cell-surface marker identified as a gastric CSC marker is the CD71 transferrin receptor. In this case, it was demonstrated that the CD71^–^ subpopulation from the MKN-1 GC cell line displayed CSC features, contrary to CD71^+^ cells. The CD71^–^ cells were more resistant to 5-FU than CD71^+^, had higher tumorigenic ability and were mostly present in the invasive front of the tumor (Ohkuma et al., 2012). The cell-surface glycoprotein CD90 (Thy-1) appeared as a potential gastric CSC marker since it was capable of identifying a small population with *in vivo* tumorigenic and self-renewal ability (Jiang J. et al., 2012). Additionally, 25% of the gastric primary tumors possessed higher expression of erb-b2 receptor tyrosine kinase 2 (HER2), which was correlated with the higher expression of CD90 (Jiang J. et al., 2012). CD133 (prominin-1), a pentaspan transmembrane glycoprotein, is described as a gastric CSC marker due to the fact that its expression is positively correlated with tumor aggressiveness in GC patients (Fukamachi et al., 2011; Lee et al., 2012; Wakamatsu et al., 2012; Hashimoto et al., 2014; Nosrati et al., 2014). Zhao et al. showed that the frequency of CD133^+^ in gastric primary tumors samples was higher than CD133^–^ cells and CD133 was associated with poor prognosis in GC (Zhao et al., 2010). Also, spheroid cells from GC cell lines and primary GC tissues presented CD133 expression and displayed several features of CSCs (Zhang X. et al., 2016). New cell-surface markers have emerged in the study of gastric CSCs and demonstrated to be able to mark a small population in GC with stem-like features, specifically Lgr5 (leucine-rich repeat-containing G-protein coupled receptor 5) and CXCR4 (C-X-C chemokine receptor type 4) also known as CD184 (Fujita et al., 2015; Gong et al., 2016). Also, the intracellular enzyme aldehyde dehydrogenase (ALDH) has been used to identify gastric CSCs (Zhi et al., 2011; Wakamatsu et al., 2012). Zhi et al. (2011) were able to divide NCI-N87 and SNU-1 GC cell lines into ALDH^+^ and ALDH^–^ cells. The ALDH^+^ cells presented CSC features such as higher levels of SOX2, NANOG and Nestin, formed more sphere-like structures and had higher resistance to 5-FU and cisplatin (Zhi et al., 2011). They also showed that ALDH^+^ cells were more sensitive to salinomycin, a drug proposed to target CSCs (Zhi et al., 2011).

### Colorectal Cancer

Several specific cell-surface markers were used to identify colorectal CSCs (Todaro et al., 2010; Vaiopoulos et al., 2012; Rassouli et al., 2016; Wahab et al., 2017; Zhou et al., 2017; Boesch et al., 2018; Munro et al., 2018; Parizadeh et al., 2019; van der Heijden and Vermeulen, 2019). CD133 was shown to be a robust CSC-surface marker in CRC (O’Brien et al., 2007; Ricci-Vitiani et al., 2007; Akbari et al., 2020). Positive expression of CD133 was, for the first time, associated with a significantly worse survival and poorer clinical response to 5-FU-based chemotherapy in CRC patients (Ong et al., 2010). CD44 is also a valid marker of colorectal CSCs (Du et al., 2008). In combination with EpCAM it has already been considered a better strategy, when compared to CD133 (Dalerba et al., 2007b). Furthermore, CD166 (another transmembrane glycoprotein) could be used to co-purify CSCs (Dalerba et al., 2007b). More recently, colonospheres and chemoresistant CRC cells were found to be enriched with CD133 and CD44 (Huang et al., 2015). A strategy that combines CD133 with CD44, seems to be more reliable to isolate colorectal CSCs from both cell lines and primary tumors (Abbasian et al., 2019). Horst et al. (2009) showed that CD133 may have a better prognostic capacity *per se*, but the combination of CD133, CD44, and CD166 markers may stratify better the risk of CRC. Lgr5 positivity identifies human colorectal CSCs and is a prognostic factor for CRC (Kemper et al., 2012; Jiang Y. et al., 2016). Lgr5 overexpression has been shown to be important in CRC progression, as a result of its role in potentiating the Wnt signaling pathway (Carmon et al., 2012). de Sousa e Melo et al. (2017) have shown that Lgr5^+^ CSCs are crucial for the formation of liver metastases, while in the primary tumor Lgr5^–^ cells proliferate and are capable of originating Lgr5^+^ cells, assuring a rapid tumor growth when treatment ends. Lgr5 combined with CD44 and EpCAM can further improve the identification of CSCs in CRC (Leng et al., 2018). Vermeulen et al. (2008) have shown that spheroid cultures of colon CSCs express CD133, CD166, CD44, CD29, CD24, Lgr5, and nuclear *β*-catenin, all of which described markers of the CSC population. Also, CD200 showed a role as a CSC marker in colon cancer, and CD49f was associated with tumor cell invasion and metastasis in colon cancer working as an important marker for identifying colorectal CSCs (Robertson et al., 2009; Haraguchi et al., 2013; Zhang S.S. et al., 2016). ALDH1 also appears as a specific marker for colonic CSCs and as an independent prognostic factor for patients with CRC (Huang et al., 2009; Lugli et al., 2010; Kahlert et al., 2012; Goossens-Beumer et al., 2014; Zhou et al., 2017; Munro et al., 2018). In patients with stage II-III rectal cancer that received radio-chemotherapy, ALDH1 expression was correlated with poor prognosis, cancer relapse and metastasis (Huang et al., 2009; Deng et al., 2014).

## Transcription Factors as Potential CSC Markers

One of the challenges to target CSCs is to identify specific markers as well as to uncover targetable molecular features associated with their phenotype. It is known that normal stem cells and CSCs share core stemness signaling pathways such as Notch, Hedgehog, WNT/*β*-catenin and JAK/STAT, that have pivotal roles in maintaining stem cell properties and regulating their transcriptional program (Chen K. et al., 2013). SOX2, OCT4, KLF4, and NANOG are some of the key TFs known to promote stemness by upregulating genes involved in self-renewal and pluripotency, while suppressing genes involved in differentiation (Young, 2011; Tang et al., 2015; Buczek et al., 2018). Key stem cell TFs like SOX2, OCT4, and NANOG have been proven to be overexpressed in CSCs. For that reason, fluorescence reporter systems driven by portions of promoters where these proteins bind were developed to allow CSCs to be labeled and tracked in various types of cancer. These reporter systems seem to be a powerful tool to identify and study CSCs more efficiently than cell-surface markers (Saygin et al., 2016). Tang et al. (2015) developed a flexible lentiviral-based reporter system (SORE6-GFP) that allows direct visualization of CSCs based on SOX2 and OCT4 expression. By using this novel reporter system, our group was able to isolate gastric CSCs from two phenotypically different GC cell lines (AGS and Kato III) (Pádua et al., 2020). Using the same principle, other authors have been developing similar systems to identify and characterize CSCs in a variety of solid tumors. Buczek et al. (2018) established a method using a lentiviral construct carrying the promoter of NANOG to identify prostate CSCs and more recently, Ghanei et al. (2020) introduced a similar approach for the isolation of CSCs in a breast cancer cell line based on the single expression of OCT4. Although the use of cell-surface markers is the most trusted approach to detect these cells, several studies demonstrated that they lack specificity and cannot be used for real time assessment of CSCs behavior, which could give new insights about their properties and possible targets (Tang et al., 2015). Growing evidences support that specific TFs overexpressed in normal gastrointestinal stem cells may contribute to the self-renewal characteristics of CSCs in GC and CRC and are related with patient prognosis (Hadjimichael et al., 2015; Zhao et al., 2017). This makes them a powerful tool in CSCs identification and study.

### SOX2 (SRY-Box Transcription Factor 2)

SOX2 is a master regulator that belongs to the family of high-mobility group TFs. It plays many roles throughout development and cell differentiation in normal tissues, namely in mammalian embryogenesis, morphogenesis and homeostasis of the foregut-derived epithelia of the esophagus, lung and trachea (Avilion et al., 2003; Sarkar and Hochedlinger, 2013). SOX2 role in stemness was strengthen with Takahashi and Yamanaka’s findings when reprogramming mouse embryonic fibroblasts into induced pluripotent stem (iPS) cells, by introducing SOX2 along with OCT3/4, c-Myc and KLF4 (Takahashi and Yamanaka, 2006). In cancer, SOX2 has increasingly been associated with a CSC phenotype in several tumors (Wuebben and Rizzino, 2017; Takeda et al., 2018). In GC, SOX2 role is still controversial: some authors associate its high expression with a more aggressive phenotype, poor prognosis and worse response to therapy whereas others have shown the opposite (Hütz et al., 2013; Camilo et al., 2014; Carrasco-Garcia et al., 2016; Zhang X. et al., 2016; Wuebben and Rizzino, 2017; Basati et al., 2020; Pádua et al., 2020). Our group identified subpopulations of gastric CSCs in two human cell lines based on the expression of SOX2 and showed that SORE6^+^ cells presented CSCs properties, including higher proliferation and ability to form gastrospheres, enhanced *in vivo* tumorigenesis and increased resistance to 5-FU (Pádua et al., 2020). Additionally, Hütz et al. (2013) observed that inhibition of SOX2 resulted in reduced cell proliferation and migration, increased apoptosis, changes in cell cycle and reduced tumorigenic potential of cells *in vivo*. Similar results were observed *in vivo*, where suppression of SOX2 resulted in reduced tumor growth and decreased tumorigenicity (Tian et al., 2012; Hütz et al., 2013). In CRC, SOX2 overexpression has been associated with tumor progression and disease recurrence and SOX2 *de novo* expression was associated with poorly differentiated and more invasive tumors and poor patient overall survival (Lundberg et al., 2014; Lundberg et al., 2016). Takeda et al. (2018) have shown that SOX2^+^ cells developed chemoresistance to oxaliplatin and 5-FU, exhibiting typical asymmetric cell division and higher CSC markers expression. They concluded that colon cancer cells expressing SOX2 behave like CSCs and are therefore associated with poor prognosis (Lundberg et al., 2016; Takeda et al., 2018). Taken together, these findings indicate that SOX2 has a critical role in several aspects of CSCs biology.

### OCT4 (POU Class 5 Homeobox 1)

OCT4, a homeodomain TF of the Pit-Oct-Unc family, is expressed in embryonic stem cells, where it maintains stem cell-like properties, and in adult stem cells, being involved in their proliferation and differentiation (Loh et al., 2006; Han et al., 2014). Abnormal expression of OCT4 has been observed in different tumor types, including GC and CRC (Dai et al., 2013; Basati et al., 2020). Studies report that its expression is positively correlated with more aggressive and metastatic tumors, as well as with poorer overall prognosis (Zhang X. et al., 2016; Basati et al., 2020). Chen and collaborators demonstrated that overexpression of OCT4 in GC cells led differentiated cancer cells to become undifferentiated and acquiring self-renewal capacity (Chen et al., 2009) and another study reported that downregulation of OCT4 induced differentiation in GC cells (Tai et al., 2005). Additionally, Zhang X. et al. (2016) demonstrated a p-ERK mediated positive feedback loop between the cell surface marker CD44 and OCT4, responsible for sustaining gastric CSCs properties. OCT4 has also been reported in colon CSCs and its knockdown inhibits cell migration and invasion, suggesting it may act as a novel prognostic marker in CRC (Miyoshi et al., 2010, 2018; Dai et al., 2013; Amini et al., 2014).

### KLF4 (Kruppel Like Factor 4)

KLF4, also known as the gut-enriched Kruppel like factor (GKLF), is strongly expressed in post-mitotic and terminally differentiated epithelial tissues, along with those of the gastrointestinal tract (Cho et al., 2007; Cui et al., 2013). It is suggested that it may have an anticancer role in GC, being downregulated due to hypermethylation and loss of heterozygosity in gastric CSCs (Cho et al., 2007). Several studies have shown that KLF4 low expression is negatively associated with patient overall survival and may be a useful prognostic marker in GC patients (Li et al., 2012; Zhang et al., 2012; Hsu et al., 2013; Hashimoto et al., 2017; Zhao et al., 2020). Regarding CRC, KLF4 role is still not clear. Some studies reveal KLF4 is overexpressed in colon CSCs and its knockdown affects the stemness phenotype and decreases the cells malignant profile, while others demonstrate that loss of expression is associated with stem-like features namely formation of colonospheres, cell growth arrest, uncontrolled cell proliferation, pluripotency and self-renewal (Shie et al., 2000; Wei et al., 2006; Leng et al., 2013; Hadjimichael et al., 2015).

### NANOG (Nanog Homeobox)

NANOG was first discovered in embryonic stem cells (ESCs) and is a key TF involved in self-renewal and multipotency (Chambers et al., 2003). It is typically silenced in normal somatic cells, though abnormal expression has been reported in malignant tumors, such as GC and CRC (Lin et al., 2012; Hadjimichael et al., 2015). Previous studies demonstrate that NANOG is highly expressed in GC and significantly associated with tumor size and grade, along with decreased overall survival (Iv Santaliz-Ruiz et al., 2014; Basati et al., 2020). Although studies indicate NANOG is associated with a CSC phenotype, it remains unclear its role in CSC maintenance in GC (Iv Santaliz-Ruiz et al., 2014). With respect to CRC, NANOG overexpression has been associated with colony formation and stem cell properties, as well as worse prognosis (Ibrahim et al., 2012; Zhang et al., 2013; Hadjimichael et al., 2015).

### c-Myc (MYC Proto-Oncogene, bHLH Transcription Factor)

c-Myc is an essential TF that regulates genes that take part in biological processes such as self-renewal, differentiation, growth and metabolism (Dang, 2013; Bretones et al., 2015). Although it is one of the most commonly activated oncogenes involved in the pathogenesis of cancer, its overexpression alone is unable to induce the transformation of normal cells into tumor cells (Yang et al., 2020). The role of c-Myc in GC is less studied than in other tumor types. It has been suggested as a CSC marker in some tumors such as small-cell lung cancer, prostate cancer, neuroblastoma, glioblastoma and hematopoietic malignancies, but the expression and relevance in GC has not yet been clarified (Yang et al., 2020). Some authors associate c-Myc deregulation with poor prognostic features (Han et al., 1999; de Souza et al., 2013; Wang et al., 2016). Upregulation of c-Myc is common in 70% of CRC cases and it has been shown to have a crucial role in maintaining chemoresistance and self-renewal, being overexpressed in colon CSCs (Muzny et al., 2012; Zhang et al., 2019). Despite some controversial results, it has been shown that high expression of c-Myc is an independent poor prognostic factor in CRC (Lee et al., 2015; Wang et al., 2017).

### SOX9 (SRY-Box Transcription Factor 9)

SOX9 regulates developmental processes such as male sex determination, chondrogenesis, neurogenesis, and neural crest development (Jo et al., 2014). Also, it plays a vital role in cell fate decisions and stem cell maintenance during embryonic development and adulthood in several organs, including the gastrointestinal tract (Bastide et al., 2007; Huch and Clevers, 2011). Its role in GC is still conflicting, while some studies defend an association between lower survival and SOX9 high expression, others demonstrate poor prognosis with a decreased level of SOX9 expression (Sun et al., 2012; Santos et al., 2016; Mesquita et al., 2019). Also, both oncogenic and tumor suppressor activity of SOX9 have been implicated in CRC (Darido et al., 2008; Lu et al., 2008; Matheu et al., 2012; Prévostel and Blache, 2017). Previous studies suggest that this TF can influence tumor proliferation and progression, mainly through the regulation of the CSC pool, and could correlate with poor prognosis (Lu et al., 2008; Espersen et al., 2015; Javier et al., 2016).

### GLI1 (GLI Family Zinc Finger 1)

GLI1 is part of the Sonic Hedgehog (SHH) pathway and seems to be essential for the maintenance of cancer cells with stem like properties in both GC and CRC (Zhang X. et al., 2016; Yang et al., 2018). In GC, its expression is significantly higher in metastatic cancer tissues and is positively correlated with a more aggressive tumor phenotype (Zhang X. et al., 2016). Furthermore, it has been observed that GLI1 overexpression promotes a CSC phenotype enhancing cell proliferation, migration and therapy resistance (Dong et al., 2019; Yao et al., 2019). GLI1 also plays an important role in CSC characteristics related with aggressiveness and metastatic spread of CRC cells leading to decreased survival. Furthermore, GLI1 knockdown downregulates CD133/SOX9 expression and clonogenic ability of CRC cells, indicating this TF could be a potential marker for CSCs in CRC (Yang et al., 2018).

### STAT3 (Signal Transducer and Activator of Transcription 3)

STAT3 plays an important role in the regulation of various physiological functions, such as inflammation, proliferation and invasion, being highly expressed in gastric and colorectal CSCs (Yu H. et al., 2009; Lin et al., 2011; Hajimoradi et al., 2016; Sonbol et al., 2019). In gastric CSCs, high expression of STAT3 has been reported to be involved in stemness properties and invasive ability (Hajimoradi et al., 2016; Jiang et al., 2017; Sonbol et al., 2019). Regarding its prognostic value several studies report STAT3 activation as a marker of unfavorable outcome (Kim D.Y. et al., 2009; Deng et al., 2010, 2013; Ji et al., 2016). In CRC, STAT3 is one of the major oncogenic proteins associated with proliferation, angiogenesis, invasion and chemo-radiotherapy resistance (Lin et al., 2005, 2011; Munro et al., 2018). Also, its inhibition prevents tumor initiation, being an attractive therapeutic target for CRC (Lin et al., 2011).

### SALL4 (Spalt Like Transcription Factor 4)

As a TF, SALL4 plays essential roles in maintaining pluripotency and self-renewal of ESCs, being downregulated or silenced in differentiated cells (Zhang et al., 2006, 2015; Yang et al., 2008). SALL4 acts as an oncogene and it is associated with cancer initiation, development, and progression (Ma et al., 2006). Zhang et al. (2014) showed that the overexpression of SALL4 is associated with gastric CSC features and could be involved in the generation and maintenance of these cells. Later, Yuan et al. (2016) suggested a novel mechanism for SALL4 role in GC, showing that this TF binds to the promoter region of CD44 and activates it expression, enhancing cell proliferation, migration and invasion. Increasing evidence indicates that upregulation of SALL4 is associated with lymph node metastasis and poorer overall prognosis (Zhang et al., 2014, 2018b). In CRC, SALL4 overexpression is detected in 87% of tumor tissues and it is correlated with tumor cell metastasis to lymph nodes being associated with poor prognosis and showing its essential role in maintaining the properties of CSCs (Forghanifard et al., 2013; Zhang et al., 2015).

### β-Catenin (Catenin Beta 1)

The Wnt/*β*-catenin pathway is implicated in the regulation of the epithelial stem cell self-renewal (Behrens et al., 1996). Alone, *β*-catenin signaling has been shown as necessary for the maintenance of CSC features (Huang et al., 2007; Kanwar et al., 2010; Jiang R. et al., 2016). The dysregulation of the Wnt/*β*-catenin signaling pathway has been implicated in colon carcinogenesis and plays a critical role in regulating the growth and maintenance of colonospheres (Kolligs et al., 2002; Kanwar et al., 2010). The activation of this pathway can lead to the conversion of intestinal stem cells into CSCs, where expression levels of *β*-catenin are higher (Kanwar et al., 2010). Some studies revealed that high levels of nuclear β-catenin, in CRC patients, were associated with a poor prognosis and could be used as a biomarker for late phase CRC (Chen Z. et al., 2013). Gastric CSCs self-renewal and proliferation ability, both *in vitro* and *in vivo*, are also improved by the Wnt/*β*-catenin signaling (Mao et al., 2014; Chiurillo, 2015).

## CSCs and Tumor Microenvironment

The close interaction between CSCs and their niche is fundamental for maintaining the stemness of CSCs and tumor progression. The CSC niche, a specific tumor microenvironment, which consists of stroma, micro-vessels, hypoxic regions, cancer-associated fibroblasts (CAFs), cancer-associated mesenchymal stem cells (MSCs), tumor-associated macrophages (TAMs) and extracellular matrix, secretes soluble factors (e.g., cytokines and growth factors) that are necessary for cancer cell survival (Quante et al., 2013; Lau et al., 2017; Yang et al., 2020). Growth factors and cytokines regulate Wnt, Notch, JAK-STAT3 and other signaling pathways thereby stimulating growth, epithelial-to-mesenchymal transition (EMT), invasion, angiogenesis, metastasis and inhibiting apoptosis (Yang et al., 2020). These pathways are required for the self-renewal and maintenance of CSCs. For instance, growth factors like hepatocyte growth factor (HGF), secreted by the stromal myofibroblasts, activate the Wnt-signaling in a subset of colon cancer cells that maintain the CSC phenotype (Vermeulen et al., 2010). Another example is hypoxia, which also maintains a stem-like phenotype in CRC and GC through the increased expression of hypoxia-inducible factors (HIFs), the transcription factors HIF-1α and HIF-2α, that maintain the Wnt/*β*-catenin signaling pathway and activate stemness-related TFs such as OCT4 (Gidekel et al., 2003; Liu et al., 2008; Mazumdar et al., 2010; Yeung et al., 2011; Vadde et al., 2017). On the other hand, cancer cells also secrete growth factors and proteases to promote changes in their microenvironment (Ishimoto et al., 2014). One significant example of this crosstalk between cancer cells and the microenvironment is the secretion of cytokines like interleukin-6 (IL-6) by the cancer-associated mesenchymal stem cells that enhance the progression of CRC through the IL-6/JAK2/STAT3 signaling (Zhang et al., 2018a). The same is observed in GC through the secretion of interleukin-8 (Li W. et al., 2015). On the other hand, cancer cells mediate the production of inflammatory cytokines with pro-tumorigenic roles or the inhibition of cytokines involved in immune surveillance, altering the composition of the immune cells in the tumor microenvironment (Quante et al., 2013; West et al., 2015). Specifically, Rezalotfi et al. (2019) suggested that CSCs could alter the cytokines in the tumor microenvironment by demonstrating that the balance between suppressive regulatory T cells (Treg) and T helper cells producing IL17 (Th17) could be affected. Chaudhry et al. (2009) disclosed further that STAT3 is fundamental for the inhibition of Treg cells development and Th17 differentiation. In fact, the STAT3 transcription factor, in collaboration with NFκB, regulates the expression of these genes encoding critical cancer-promoting inflammatory mediators, establishing a crosstalk between cancer and immune cells of the microenvironment and perpetuating the effects of STAT3 activation in cancer cells (Yu et al., 2007; Grivennikov and Karin, 2010; Yang et al., 2019). However, despite the growing evidences on the interaction of gastric and colorectal CSCs with the tumor microenvironment, the specific molecules involved and their signaling pathways still need further investigation in order to design safe therapies.

## Therapeutic Approaches to Target CSCs

The therapeutic approach in GC and CRC is determined by the stage of the disease at the time of diagnosis. Patients are treated with surgery, chemotherapy and/or radiation. In GC, surgery remains the main treatment for stage I. It can also be performed at stages II and III but chemotherapy (perioperative, neoadjuvant or adjuvant) is necessary to improve overall survival of the patients. For stage IV, chemotherapy with doublet or triplet platinum/fluoro-pyrimidine combinations or capecitabine is the main treatment (Neri et al., 2007; Smyth et al., 2016). Resective surgery is the main curative treatment used in non-metastasized CRC, although neo-adjuvant treatments are also administered in rectal carcinoma (Kuipers et al., 2015). After surgery, 5-FU-based chemotherapy is used to reduce the risk of tumor recurrence and improve overall survival of patients (Dienstmann et al., 2017). Currently, the decision of giving adjuvant treatment to early-stage CRC patients is recommended to high risk patients with one or more risk factors (Kannarkatt et al., 2017). Patients with very high risk – microsatellite stable (MSS) and T4 may be considered for the addition of oxaliplatin (Labianca et al., 2013). However, in some cases of GC and CRC, after a believed efficacious treatment, the cancer reappears locally or in distant metastasis. This results from the presence of CSCs that were able to resist the therapy applied, revealing that the existence of CSCs is one of the biggest difficulties in cancer treatment (Dean et al., 2005). Thus, direct targeting of CSCs seems to be the key for tumor complete elimination. The therapy against CSCs using specific molecules should eradicate these cells while the conventional therapy eliminates the non-CSCs present in the tumor bulk. Nevertheless, this type of treatment should be administrated in combination due to the possibility of cell plasticity that facilitates the appearance of *de novo* CSCs from non-CSCs. Moreover, targeting specific TFs or signaling pathways responsible for maintaining the CSC phenotype could become novel therapies against GC and CRC. For that reason, several clinical trials are being undertaken to explore the efficacy of diverse compounds, that are capable of modulating or inactivating proteins that gastric and colorectal CSCs use to grow and survive, allowing their elimination. Additionally, it is worth to mention that several studies have been conducted to evaluate the efficacy of small molecules in targeting CSCs *in vitro* and *in vivo*, compounds capable of eliminating or reducing the CSC population that could also be part of the path in cancer therapy targeting CSCs (Gupta et al., 2009; Abetov et al., 2015; Shapiro et al., 2016; Müller et al., 2017; Park et al., 2017; Pádua et al., 2020). Table 2 lists the drugs that have been or are being investigated in clinical trials, alone or in combination with other compounds, to treat GC and CRC.

**TABLE 2:** Complete and ongoing clinical trials of therapeutic agents targeting gastric and/or colorectal CSCs, correlated signaling pathways and the transcription factor STAT3.

**Drug/Antibody**	**Targets**	**Disease/Condition**	**NCT Identifier**	**Phase**	**Routes of administration**	**Recruitment status**	**Last update**	**Sponsor**
**Immunotherapy**								
**RO5429083**	CD44	Metastatic and/or locally advanced malignant solid tumors	NCT01358903	I	Intravenous (IV)	Completed, no results posted	November 2016	Hoffmann-La Roche
**Adecatumumab** alone or in combination with FOLFOX	EpCAM	R0 resection of colorectal liver metastases	NCT00866944	II	Intravenous (IV)	Completed, no results posted	November 2011	Amgen Research (Munich) GmbH
**MCLA-158**	EGFR and Lgr5	Advanced/metastatic solid tumors, including CRC	NCT03526835	I	Intravenous (IV)	Recruiting	August 2018	Merus N.V.
**EpCAM CAR-T**	EpCAM	Relapsed or refractory EpCAM positive cancer	NCT03013712	I/II	Intravenous (IV)	Recruiting	January 2017	First Affiliated Hospital of Chengdu Medical College
**EpCAM CAR-T**	EpCAM	Advanced GC with peritoneal metastasis	NCT03563326	I	Intravenous (IV)	Recruiting	September 2018	Jian-Kun Hu
**EpCAM CAR-T**	EpCAM	GC	NCT02725125	—	Intravenous (IV)	Unknown	March 2017	Sinobioway Cell Therapy Co., Ltd.
**CART-133**	CD133	Chemotherapy refractory advanced malignancies, including CRC	NCT02541370	I/II	Intravenous (IV)	Completed, no results posted	December 2019	Chinese PLA General Hospital
**Transcription Factor inhibitors**								
**BBI608** or BNC105 in combination with nivolumab	STAT3	Metastatic CRC	NCT03647839	II	Oral and Intravenous (IV)	Recruiting	February 2019	Australasian Gastro-Intestinal Trials Group
**Danvatirsen (AZD9150)** in combination with Durvalumab (MEDI4736)	STAT3	Advanced and refractory pancreatic, non-small cell lung cancer, and mismatch repair deficient CRC	NCT02983578	II	Intravenous (IV)	Recruiting	March 2020	M.D. Anderson Cancer Center
**Napabucasin (GB201)** in combination with FOLFIRI	STAT3	Metastatic CRC	NCT03522649	III	Oral and Intravenous (IV)	Recruiting	June 2019	1Globe Health Institute LLC
**TTI-101**	STAT3	Advanced cancers, including gastric adenocarcinoma and CRC	NCT03195699	I	Oral	Recruiting	February 2020	Tvardi Therapeutics, Incorporated
**Artesunate**, prior to surgery	*β*-catenin	Stage II/III CRC	NCT02633098	II	Oral	Recruiting	April 2019	St George’s, University of London
**DKN-01** in combination with atezolizumab	DKK1	GC	NCT04166721	I/II	Intravenous (IV)	Recruiting	February 2020	Royal Marsden NHS Foundation Trust
**ETC-1922159** in combination with pembrolizumab	PORCN	Advanced solid tumors	NCT02521844	I	Oral and Intravenous (IV)	Active, not recruiting	October 2019	EDDC (Experimental Drug Development Centre), A*STAR Research Entities
**Foxy-5**	Wnt-5A	CRC	NCT02020291	I	Intravenous (IV)	Completed, no results posted	February 2016	WntResearch AB
**Foxy-5**	Wnt-5A	Metastatic breast, colon or prostate cancer	NCT02655952	I	Intravenous (IV)	Completed, no results posted	December 2018	WntResearch AB
**Foxy-5** as neo-adjuvant therapy	Wnt-5A	Colon cancer	NCT03883802	II	Intravenous (IV)	Recruiting	April 2019	WntResearch AB
**Genistein** in combination with FOLFOX or FOLFOX-Avastin	tyrosine kinase and topoisomerase-II	Metastatic CRC	NCT01985763	I/II	Oral and Intravenous (IV)	Completed, has results	May 2019	Sofya Pintova
**LGK974**	PORCN	Malignancies dependent on Wnt ligands, including BRAF mutant CRC	NCT01351103	I	Oral	Recruiting	February 2020	Novartis Pharmaceuticals
**LGK974** in combination with LGX818 and cetuximab	PORCN	BRAF-mutant metastatic CRC and Wnt pathway mutations metastatic CRC	NCT02278133	I/II	Oral	Completed, no results posted	October 2017	Array BioPharma
**MCLA-158**	EGFR and LGR5	Advanced/metastatic solid tumors, including CRC	NCT03526835	I	Intravenous (IV)	Recruiting	August 2018	Merus N.V.
**Mesalazine**	induces the expression of μ-protocadherin	CRC	NCT02077777	II	Oral	Completed, no results posted	December 2016	SOFAR S.p.A.
**OMP-18R5 (Vantictumab)**	Wnt receptor	Solid tumors	NCT01608867	I	Intravenous (IV)	Completed, no results posted	September 2016	OncoMed Pharmaceuticals, Inc.
**OMP-54F28 (Ipafricept)**	Wnt receptor	Solid tumors	NCT01608867	I	Intravenous (IV)	Completed, no results posted	July 2017	OncoMed Pharmaceuticals, Inc.
**Hedgehog (Hh) pathway inhibitors**								
**BMS-833923 (XL139)**	Smoothened	Advanced or metastatic solid tumors	NCT01413906	I	Oral	Completed, no results posted	June 2013	Bristol-Myers Squibb
**BMS-833923** in combination with cisplatin and capecitabine	Smoothened	Metastatic gastric, gastroesophageal, or esophageal adenocarcinomas	NCT00909402	I	Oral and Intravenous (IV)	Completed, no results posted	June 2013	Bristol-Myers Squibb
**LDE255 (Sonidegib)** in combination with BKM120 (Buparlisib)	Smoothened (and PI3K)	Advanced solid tumors	NCT01576666	I	Oral	Completed, no results posted	February 2016	Novartis Pharmaceuticals
**LEQ-506**	Smoothened	Advanced solid tumors	NCT01106508	I	Oral	Completed, no results posted	February 2020	Novartis Pharmaceuticals
**Vismodegib** in combination with standard chemotherapy	Smoothened	Advanced GC or gastroesophageal junction cancer	NCT00982592	II	Oral and Intravenous (IV)	Completed, has results	January 2016	National Cancer Institute (NCI)
**Vismodegib (GDC-0449)** with concurrent chemotherapy and bevacizumab as first-line therapy	Smoothened	Metastatic CRC	NCT00636610	II	Oral	Completed, has results	June 2017	Genentech, Inc.
**NOTCH pathway inhibitors**								
**BMS-906024**	Pan-Notch	Advanced or metastatic solid tumors	NCT01292655	I	Intravenous (IV)	Completed, no results posted	January 2020	Bristol-Myers Squibb
**CB-103**	Pan-Notch	Advanced or metastatic solid tumors, including CRC	NCT03422679	I/II	Oral	Recruiting	May 2019	Cellestia Biotech AG
**LY3039478 (Crenigacestat)**	Pan-Notch	Advanced solid tumors	NCT02836600	I	Oral	Active, not recruiting	December 2019	Eli Lilly and Company
**LY3039478 (Crenigacestat)** in combination with other anticancer agents	Pan-Notch	Advanced or metastatic solid tumors	NCT02784795	I	Oral and Intravenous (IV)	Completed, no results posted	March 2020	Eli Lilly and Company
**LY900009**	γ-Secretase	Advanced cancer	NCT01158404	I	Oral	Completed, has results	August 2019	Eli Lilly and Company
**MEDI0639**	DLL4	Advanced solid tumors	NCT01577745	I	Intravenous (IV)	Completed, has results	May 2017	MedImmune LLC
**REGN421 (SAR153192; Enoticumab)**	DLL4	Advanced solid malignancies	NCT00871559	I	Intravenous (IV)	Completed, no results posted	March 2014	Regeneron Pharmaceuticals
**RO4929097**	γ-Secretase	Recurrent and metastatic CRC	NCT01116687	II	Oral	Completed, has results	May 2014	National Cancer Institute (NCI)
**RO4929097** in combination with capecitabine	γ-Secretase	Refractory solid tumors	NCT01158274	I	Oral	Completed, no results posted	November 2014	National Cancer Institute (NCI)
**RO4929097** in combination with cediranib maleate	γ-Secretase	Advanced solid tumors including CRC	NCT01131234	I	Oral	Completed, no results posted	December 2014	National Cancer Institute (NCI)
**PI3K/AKT/mTOR pathway inhibitors**								
**Acetylsalicylic acid (aspirin)**	—	CRC stage I-III with mutations in the PI3K signaling pathway	NCT02647099	III	Oral	Recruiting	August 2019	Anna Martling
**Acetylsalicylic acid (aspirin)**	—	Resected colon cancer with PI3K mutation stage II or III high risk	NCT02945033	III	Oral	Recruiting	October 2019	University Hospital, Rouen
**Acetylsalicylic acid (aspirin)**	—	Dukes C and high-risk dukes B CRCs	NCT00565708	III	Oral	Recruiting	September 2019	National Cancer Centre, Singapore
**Acetylsalicylic acid (aspirin) and metformin**	—	Stage I–III CRC patients	NCT03047837	II	Oral	Recruiting	February 2019	Ente Ospedaliero Ospedali Galliera
**AZD2014 (Vistusertib)**	mTORC1 and mTORC2	RICTOR amplified GC	NCT03061708	II	Oral	Terminated (lack of efficacy)	May 2019	Samsung Medical Center
**AZD2014 (Vistusertib)**	mTORC1 and mTORC2	TSC1/2 mutated or TSC1/2 null GC	NCT03082833	II	Oral	Terminated (lack of efficacy)	May 2019	Samsung Medical Center
**AZD5363** in combination with paclitaxel	AKT	Advanced gastric adenocarcinoma	NCT02451956	II	Oral and Intravenous (IV)	Completed, no results posted	December 2019	Samsung Medical Center
**AZD8186** in combination with paclitaxel	PI3K β/δ	Advanced GC	NCT04001569	I/II	Oral and Intravenous (IV)	Recruiting	June 2019	Seoul National University Bundang Hospital
**BKM120 (Buparlisib)** in combination with LDE255 (Sonidegib)	PI3K and Smoothened	Advanced solid tumors	NCT01576666	I	Oral	Completed, no results posted	February 2016	Novartis Pharmaceuticals
**BYL719 (Alpelisib)** in combination with AUY922	PI3K	Advanced or metastatic GC	NCT01613950	I	Oral and Intravenous (IV)	Completed, no results posted	February 2020	Novartis Pharmaceuticals
**BYL719 (Alpelisib)** in combination with LGX818 (Encorafenib) and Cetuximab	BRAF, EGFR and PI3K	in BRAF Mutant Metastatic CRC	NCT01719380	I/II	Oral and Intravenous (IV)	Completed, no results posted	April 2019	Array BioPharma
**Cabozantinib**	Multikinases	Refractory metastatic CRC	NCT03542877	II	Oral	Active, not recruiting	April 2019	Academic Thoracic Oncology Medical Investigators Consortium
**CB-839 (Telaglenastat)** in combination with capecitabine	Glutaminase	Solid tumors and fluoropyrimidine resistant PIK3CA mutant CRC	NCT02861300	I/II	Oral	Recruiting	March 2020	Case Comprehensive Cancer Center
**Copanlisib** in combination with anti-PD-1 antibody Nivolumab	PI3K	Relapsed/refractory solid tumors, mismatch-repair proficient (MSS) CRC	NCT03711058	I/II	Intravenous (IV)	Recruiting	July 2019	Sidney Kimmel Comprehensive Cancer Center at Johns Hopkins
**DS-7423**	PI3K and mTOR	Advanced solid malignant tumors	NCT01364844	I	Oral	Completed, no results posted	February 2014	Daiichi Sankyo, Inc.
**Ipatasertib (GDC-0068)** in combination with oxaliplatin, 5-FU and leucovorin	AKT	Advanced or metastatic GC or gastroesophageal junction cancer	NCT01896531	II	Oral and Intravenous (IV)	Active, not recruiting	February 2020	Genentech, Inc.
**MK-2206**	AKT	Advanced GC or gastroesophageal junction cancer	NCT01260701	II	Oral	Completed, has results	January 2016	National Cancer Institute (NCI)
**MK-2206**	AKT	CRC that is metastatic or locally advanced and cannot be removed by surgery	NCT01802320	II	Oral	Completed, has results	September 2019	National Cancer Institute (NCI)
**MK-2206** in combination with AZD6244 (Selumetinib)	AKT and MEK	Advanced CRC	NCT01333475	II	Oral	Completed, has results	September 2015	National Cancer Institute (NCI)
**Neratinib** in combination with Trastuzumab or with Cetuximab	HER2 and EGFR	KRAS/NRAS/BRAF/PIK3CA wild-type metastatic CRC by HER2 status	NCT03457896	II	Oral	Recruiting	November 2019	NSABP Foundation Inc
Pembrolizumab (MK-3475) combined with **INCB050465** or with Itacitinib (INCB039110)	JAK1 and PI3K-delta	Advanced solid tumors, including CRC	NCT02646748	I	Oral	Active, not recruiting	December 2019	Incyte Corporation
**PX-866 (Sonolisib)** in combination with Cetuximab	PIP3	Incurable metastatic CRC	NCT01252628	I/II	Oral	Completed	May 2018	Cascadian Therapeutics Inc.
**RAD001 (Everolimus)**	TOR serine-threonine kinases	Previously treated unresectable or metastatic esophageal cancer or GC	NCT00985192	II	Oral	Completed, has results	February 2020	Translational Oncology Research International
**RAD001 (Everolimus)**	TOR serine-threonine kinases	Advanced GC	NCT00519324	II	Oral	Completed, no results posted	November 2015	Novartis Pharmaceuticals
**RAD001 (Everolimus)** in combination with AV-951(Tivozanib)	TOR serine-threonine kinases and VEGFRs 1, 2, and 3	Gastrointestinal cancer	NCT01058655	I/II	Oral	Completed, has results	April 2017	Dana-Farber Cancer Institute
**RAD001 (Everolimus)** in combination with Capecitabine and Oxaliplatin	TOR serine-threonine kinases	Advanced GC	NCT01049620	I	Oral and Intravenous (IV)	Completed, no results posted	January 2020	Asan Medical Center
**RAD001 (Everolimus)** in combination with cisplatin and HDFL (high-dose 5-FU and leucovorin)	TOR serine-threonine kinases	Advanced GC	NCT00632268	II	Oral and Intravenous (IV)	Completed, no results posted	August 2013	National Taiwan University Hospital
**SAR245409 (Voxtalisib)** in combination with MSC1936369B (pimasertib)	PI3K, MTOR, MEK1 and 2	Advanced or metastatic solid tumors (GC not included)	NCT01390818	I	Oral	Completed, has results	March 2017	EMD Serono
**Serabelisib** in combination with Canagliflozin	PI3Kα and SGLT2	Advanced solid tumors	NCT04073680	I/II	Oral	Not yet recruiting	February 2020	Petra Pharma
**SF1126**	PI3K	Solid tumors	NCT00907205	I	Intravenous (IV)	Completed, no results posted	June 2013	Semafore Pharmaceuticals
**Trametinib** in combination with Trifluridine and Tipiracil Hydrochloride	MEK	Chemotherapy-resistant RAS-mutated (PIK3CA/PTEN-Wild-Type) metastatic CRC	NCT03317119	I	Oral	Recruiting	January 2020	City of Hope Medical Center

*GC corresponds to gastric cancer and CRC to colorectal cancer. FOLFOX corresponds to a chemotherapy regimen with folinic acid (leucovorin), fluorouracil (5-FU) and oxaliplatin (Eloxatin) and FOLFIRI corresponds to a chemotherapy regimen with folinic acid (leucovorin), fluorouracil (5-FU) and irinotecan (Camptosar).*

### Immunotherapy

Cancer immunotherapy has emerged as a potential tool for cancer treatment (Farkona et al., 2016). Several immunotherapeutic strategies have been developed including cancer vaccines, oncolytic viruses, monoclonal antibodies or recombinant proteins, chimeric antigen receptor T cell (CAR-T) cells and other cellular therapies, lymphocyte-activating cytokines and checkpoint inhibitors (Riley et al., 2019). Immunotherapy aims to improve the immune system response against cancer cells through natural mechanisms (Riley et al., 2019). Therefore, it can be used to target cancer cells and also CSCs in the tumor microenvironment (Badrinath and Yoo, 2019). Many immunotherapeutic agents targeting CSCs have been tested in clinical trials (Menon et al., 2016). Monoclonal antibodies that specifically target CSC surface biomarkers have been used in gastric and colorectal cancer. Ongoing there is a Phase I study of RO5429083, a monoclonal antibody against CD44 in patients with metastatic and/or locally advanced malignant solid tumors (NCT01358903). Also used were EpCAM antibodies, such as edrecolomab, that was tested in patients with resected stage II adenocarcinoma of the colon (Niedzwiecki et al., 2011) and adecatumumab, that is being tested in a Phase II study to evaluate efficacy and safety, alone or with FOLFOX, in metastasized CRC (NCT00866944). A Phase I dose finding study is evaluating the bispecific antibody targeting EGFR and Lgr5 (MCLA-158) in metastatic CRC and other advanced solid tumors (NCT03526835). In addition, CAR-T cell therapies, have been developed to target CSCs in GC and CRC. There are three Phase I or II clinical trials in GC using CAR-T cells targeting EpCAM (NCT03013712, NCT03563326, and NCT02725125), one of them consisting in the intraperitoneal infusion in advanced GC with peritoneal metastasis. NCT03013712 includes patients with colon cancer. Moreover, there is a Phase I/II clinical study of CAR-T cells targeting CD133 in relapsed and/or chemotherapy refractory malignancies including CRC (NCT02541370).

### Targeting the Transcription Factor STAT3

From the list of TFs strongly associated with CSC phenotype in GC and CRC, STAT3 became crucial as a molecular target for cancer therapy because napabucasin (BBI608), the first-in-class cancer stemness (CSCs) inhibitor that works by targeting STAT3, effectively blocks cancer relapse and metastasis in xenografted human cancers (Li Y. et al., 2015; Zhang Y. et al., 2016). Napabucasin is a naturally occurring drug orally administered. It inhibits CSC self-renewal and induces apoptosis in CSCs by targeting CSC signaling pathways (STAT3, NANOG and *β*-catenin) in GC (Bekaii-Saab and El-Rayes, 2017). In CRC, napabucasin has been investigated in several studies. A phase I/II clinical trial investigated napabucasin with other standard therapeutic treatments for advanced gastrointestinal malignancies (Bendell et al., 2017). Another study – CanStem303C – a randomized phase III clinical trial done in adult patients with previously treated metastatic CRC evaluated napabucasin in combination with FOLFIRI (Grothey et al., 2017). Napabucasin monotherapy has been reported in a published phase III trial. The CO.23 trial evaluated the efficacy of napabucasin monotherapy versus placebo in metastasized CRC, which failed to demonstrate a significant difference in the napabucasin group survival. However, in a pre-specified biomarker analysis, phosphorylated STAT3 (pSTAT3)-positive patients experienced a significant survival benefit from napabucasin over placebo (Jonker et al., 2018; Sonbol et al., 2019). Another ongoing trial involving STAT3 inhibition is the phase II trial MODULATE (NCT03647839) which specifically aims to study the modulation of the tumor microenvironment using either vascular disrupting agents (BNC105) or STAT3 inhibition (BBI608), in synergy with an immune checkpoint protein (PD1) inhibitor (nivolumab). This trial is recruiting microsatellite stable, refractory CRC cases.

### Targeting the Wnt/β-Catenin Signaling Pathway

Wnt/*β*-catenin signaling pathway is a major regulator of normal intestinal development and its over-activation behaves as a hallmark of CRC, being particularly significant in drug resistance and stemness maintenance of colorectal CSCs (Takebe et al., 2011; Basu et al., 2016). The activation of the Wnt/*β*-catenin signaling pathway is associated with poor prognosis (Janssen et al., 2006). It results mostly from the accumulation of mutations in the APC tumor suppressor gene, oncogenic KRAS-signaling pathway, *β*-catenin and p53. Mutations that do not allow the formation of the APC/Axin/GSK3*β* destruction complex result in the accumulation and nuclear translocation of β-catenin that binds to transcription factors of T cell factor family (TCF4) and activates target genes like c-Myc, cyclinD1 and survivin, some involved in maintaining stemness (Myant et al., 2013; Lee et al., 2015; Zhou et al., 2017). It has been shown that loss of APC in CRC triggered the expression of a Rac1 GTPase via the induced expression of c-Myc, necessary to intestinal stem cell proliferation and CRC initiation (Myant et al., 2013). On the other hand, p53 may affect the outcome of Wnt signaling in CRC development (Voorneveld et al., 2015), having a role in the acquisition of pluripotency during reprogramming (Takahashi and Yamanaka, 2006; Krizhanovsky and Lowe, 2009). The Wnt signaling pathway is also dysregulated in GC (Chiurillo, 2015). However, the involvement and mechanisms are not yet as fully understood as in CRC. A number of studies suggest β-catenin and APC as driver genes, revealing somatic mutations in both genes that might have relevance in GC (Horii et al., 1992; Nakatsuru et al., 1993; Woo et al., 2001; Clements et al., 2002; Zhang and Xue, 2008). Genomic analysis of several gastric primary tumors disclosed that Wnt/*β*-catenin, together with NF-κB and proliferation/stem cell pathways, were deregulated in more than 70% of the primary tumors. Patient stratification by combinations of these oncogenic pathways revealed to be a great tool for GC clinical behavior assessment (Ooi et al., 2009). Many molecules have been used to target the Wnt/*β*-catenin in gastrointestinal CSCs (Chiurillo, 2015; Parizadeh et al., 2019; Patel et al., 2019; Yang et al., 2020). Genistein, a soy-derived compound, was tested in a Phase I/II research trial, in combination with FOLFOX or FOLFOX-Bevacizumab, where it was demonstrated to be well tolerated by patients and may have improved efficacy in the treatment of metastatic CRC (Pintova et al., 2019). *In vitro* and *in vivo* studies have shown that Genistein affects mainly Wnt/*β*-catenin and PI3K/AKT pathways (Su et al., 2003; Kim et al., 2005; Zhang and Chen, 2011; Wang et al., 2012).

### Targeting the Hedgehog Signaling Pathway

The dysregulation of Hedgehog (Hh) signaling pathway has been reported as another main cause of CSCs self-renewal and chemoresistance, being associated with poor clinical outcome in patients with GC or CRC (Ma et al., 2005; Varnat et al., 2009; Takebe et al., 2011; Usui et al., 2018). In this pathway, target gene expression is predominantly regulated by the Smoothened (SMO) protein but GLI inhibitors are also used (Rimkus et al., 2016; Didiasova et al., 2018). In GC cells, SMO regulates nuclear translocation of GLI-1 that in turn promotes transcription of target genes, such as CD44 (Yoon et al., 2014). Yoon et al. (2014) showed that in AGS, MKN-45, and NCI-N87 GC cell lines, the Hh pathway inhibition using SMO shRNA or small-molecule inhibitors significantly decreased spheroid formation ability and tumor growth. Vismodegib (GDC-0449), the first Hh pathway inhibitor used in cancer research, is currently undergoing Phase II trials in advanced GC and in metastatic CRC (Gupta et al., 2010; Berlin et al., 2013). Examination of tumor samples revealed that Vismodegib has not increased progression-free or overall survival as a whole, but only in a limited subset of patients with high CD44 expression (Cohen et al., 2013; Yoon et al., 2014). Disappointingly, these treatments with Vismodegib, did not increase progression-free survival in CRC patients (Low and de Sauvage, 2010; McMillan and Matsui, 2012). When the SMO inhibitor AY9944 was used in combination with the GLI-1 inhibitor GANT61, there was an increased response to anti-cancer drugs in tumor organoids and a decreased capacity to form colonies in SW480 and HCT116 CRC cells (Usui et al., 2018). These results indicate that this strategy might be worthwhile in CRC.

### Targeting the Notch Signaling Pathway

The Notch signaling pathway is one of the most activated signaling pathways in cancer, namely in GC and CRC, and promotes metastization (Du et al., 2014; Hayakawa et al., 2019). It has a key role in the maintenance and differentiation of CSCs (Quail et al., 2012; Lu et al., 2013; Yang et al., 2020). In GC and CRC, the expression of Notch1 or Jagged1 is associated with poor prognosis (Yeh et al., 2009; Kang et al., 2012; Jackstadt et al., 2019; Kim et al., 2019; Mohamed et al., 2019). In CRC, Notch1 is associated with more aggressive subtypes, recruiting neutrophils to drive metastasis (Jackstadt et al., 2019). Due to the fact that the Notch pathway is related to CSC self-renewal and angiogenesis, targeting this pathway became a potential anti-CSC therapeutic approach (Venkatesh et al., 2018). Strategies used to inhibit Notch signaling include γ-secretase inhibition (GSI), Notch receptor (e.g., Notch1, Notch2, and Notch3) or ligand (e.g., Jagged1 and Jagged2) antibodies and combination therapy with inhibitors of other pathways. The inhibition of Notch receptors using two GSIs has allowed to disclose the importance of Notch pathway in the growth and survival of GC cells (Brzozowa et al., 2013). Furthermore, GSIs lead to the induction of apoptosis and inhibition of tumor-sphere formation of CD44^+^ gastric CSCs (Barat et al., 2017). However, it is important to mention that GSIs do not only target Notch-related proteins but also proteases involved in numerous cellular processes, which could originate adverse effects *in vivo* (Shih Ie and Wang, 2007; Wang et al., 2010; Brzozowa et al., 2013). Nevertheless, various clinical trials have been performed to evaluate the efficacy of targeting the Notch signaling pathway in GC and CRC (Table 2). RO4929097, a selective GSI, showed good anti-tumor activity in preclinical and early trials but it was not good enough for metastatic CRC (Luistro et al., 2009; Strosberg et al., 2012; Tolcher et al., 2012). Combinations of RO4929097 with other drugs in advanced solid tumors, including CRC, were well tolerated and presented some clinical benefit (Sahebjam et al., 2013; LoConte et al., 2015). LY900009 is another GSI tested in advanced cancers, including those of gastrointestinal tract. It is currently in a phase I trial and revealed a safety profile, with the majority of patients experiencing low-grade gastrointestinal adverse effects (Pant et al., 2016). Furthermore, it was demonstrated to have an inhibitory effect on Notch signaling pathway, inducing goblet cell differentiation and increased mucin production, similarly to that observed in rats (Pant et al., 2016). Moreover, MEDI0639 is a monoclonal antibody that specifically binds to DLL4 and prevents its interaction with Notch receptors, thereby inhibiting Notch-mediated signaling and target gene transcription, which may block tumor angiogenesis and eventually tumor cell growth (Ishigami et al., 2013). A phase I study in advanced solid tumors demonstrated that MEDI0639 is well tolerated and preliminary results show evidence of antitumor activity (Falchook et al., 2015).

### Targeting the PI3K/AKT/mTOR Signaling Pathway

The PI3K/AKT/mammalian target of rapamycin (mTOR) signaling pathway is typically abnormally activated in many carcinomas (Michl and Downward, 2005; Johnson et al., 2010; Narayanankutty, 2019). It is thought to be crucial in angiogenesis, cell proliferation, metabolism, survival, metastasis and drug resistance (Cantley, 2002; Edinger and Thompson, 2002; Fingar et al., 2004; Al-Batran et al., 2012; Tapia et al., 2014). AKT is commonly overexpressed in tumors and plays an important role in the metabolic reprogramming of cancer (Yap et al., 2011; Iida et al., 2013). Although the PI3K/AKT/mTOR pathway has been extensively studied, there are few studies in CSCs. In GC and CRC, activation of mTOR appears to cause tumor progression and poor patient survival (Lang et al., 2007; Murayama et al., 2009; Xiao et al., 2009; Yu G. et al., 2009; An et al., 2010). Thus, its inhibition seems to be fundamental for GC and CRC therapy. A phase II study using RAD001 (everolimus) in previously treated unresectable or metastatic esophageal cancer or GC was performed over the rational that everolimus may stop tumor growth by blocking some of the fundamental enzymes for cell growth and by blocking angiogenesis. Everolimus was well tolerated by the patients however this study displayed a strong weakness by being single-arm and non-comparative. However, mTOR suppression decreased ALDH1 activity, which is a marker of CSCs in CRC (Xia and Xu, 2015). Growth inhibition, using a dual PI3K/mTOR inhibitor, PF-04691502, was observed *in vitro* and in xenografted CRC tumors (Fang et al., 2013). Another mTOR inhibitor decreased survival and invasion of colorectal CSCs *in vitro*, and suppressed tumor growth *in vivo* (Francipane and Lagasse, 2013). The allosteric AKT inhibitor (MK-2206) led to a decrease in CSCs proliferation, and reduction of the capacity to form colonospheres *in vitro* and to initiate tumor formation *in vivo*. Mice with xenografted tumors showed a significant decrease in tumor progression. Also, MK-2206 significantly inhibited the growth of patient-derived tumorspheres (Malkomes et al., 2016). A phase II study in advanced gastric or gastroesophageal junction cancer has revealed that MK-2206, as second-line therapy, at a dose of 60 mg was well tolerated by patients and showed some modest evidence of activity, however, the overall survival (5.1 months) was lower than the study efficacy endpoint (6.5 months) (Ramanathan et al., 2015). However, there is a Phase II clinical trial with MK-2206, to study patients with previously treated colon or rectal cancer that has spread and cannot be removed by surgery, concluding that in contrast to robust preclinical data, it does not have effect in these tumors (Dasari et al., 2016). MK-2206 was also used in combination with AZD6244 (Selumetinib), a mitogen-activated protein extracellular signal-regulated kinase (MEK)1/2 inhibitor, in a Phase II trial but the level of target inhibition obtained with the maximum non-toxic dose was not the expected (Do et al., 2015). SAR245409 (Voxtalisib) was tested in a Phase I research trial in combination with another MEK inhibitor, MSC1936369B (Pimasertib), in advanced or metastatic solid tumors (Schram et al., 2018). The primary purpose of the study was to determine the maximum tolerated dose of the drug combination. The drug RAD001 (Everolimus) downregulates mTOR. A combination of RAD001, mFOLFOX-6 and Bevacizumab has been shown to be efficacious and safe in metastatic CRC (Weldon Gilcrease et al., 2019). A multicenter phase II study for patients with refractory, metastatic CRC concluded good tolerability and efficacy of Everolimus combined with Tivozanib (an oral VEGF receptor-1, -2, -3 inhibitor) with 50% of the patients having stable disease at 2 months (Wolpin et al., 2013).

## Discussion

The identification of CSCs remains a challenging task, particularly in solid tumors like GC and CRC. The use of cell surface markers as a primary tool to identify gastric and colorectal CSCs has disclosed some weaknesses and for that reason the uncovering of more reliable biomarkers must become a priority. These biomarkers should include the TFs that are required for the maintenance of gastric and colorectal CSCs phenotype, as well as components of the signaling pathways that have key roles in CSC features. This explains why TFs such as STAT3 and signaling pathways like Wnt/*β*-catenin, Hedgehog, NOTCH and PI3K/AKT/mTOR emerged as powerful targets, whose inactivation or modulation could eliminate gastric and colorectal CSCs. This fact is corroborated by several completed and ongoing clinical trials targeting these potential biomarkers in both tumor types, where some of the molecules have shown promising results. The incapacity to achieve the wanted levels of target inhibition was the major shortcoming of the clinical trials. Yet, the use of higher doses is not possible due to toxicity problems, which led to the development of combinations of drugs targeting different pathways. Furthermore, it is more advisable to measure the outcome of the treatments in terms of CSCs behavior, by assessing capacity to metastasize and re-growth after removing the drug.

## Future Perspectives

The validation of potential gastric and colorectal CSCs biomarkers and their association with GC and CRC stage is imperative to understand patient prognosis and apply a more suitable therapy. The development of a robust therapy combining CSC targets with conventional chemotherapy could be the solution to overcome resistance to anti-cancer drugs and completely eliminate cancer. Considering these issues, it is crucial that future studies further explore the role of TFs and components of signaling pathways on cancer stemness in order to develop therapies that could eradicate CSCs.

## Author Contributions

All authors listed have made a substantial, direct and intellectual contribution to the work, and approved it for publication.

## Conflict of Interest

The authors declare that the research was conducted in the absence of any commercial or financial relationships that could be construed as a potential conflict of interest.
